# Editorial: Sigma Receptors

**DOI:** 10.3389/fphar.2020.590519

**Published:** 2020-08-27

**Authors:** Ebru Aydar, Enrique J. Cobos, Tangui Maurice, Ruth D. Murell-Lagnado, Stephen T. Safrany

**Affiliations:** ^1^ Institute of Ophthalmology, University College London, London, United Kingdom; ^2^ Instituto de Investigación Biosanitaria ibs.GRANADA and Department of Pharmacology, Faculty of Medicine, University of Granada, Granada, Spain; ^3^ MMDN, Univ Montpellier, EPHE, INSERM, Montpellier, France; ^4^ School of Life Sciences, University of Sussex, Brighton, United Kingdom; ^5^ School of Medicine, RCSI-Bahrain, Adliya, Bahrain

**Keywords:** sigma-1 receptor, sigma-2 receptor, pain, cancer, ion channels, allosteric

Sigma receptors were initially described as opioid receptors (Martin et al., 1976). They are now considered neither related to other types of receptor nor to each other. While several ligands used to study these receptors [e.g., di-*O*-tolyl guanidine (DTG) and haloperidol] have similar affinities for the sigma-1 and sigma-2 receptors, these proteins show little homology at the primary amino acid level. The sigma-1 receptor was cloned some time ago (Hanner et al., 1996; Kekuda et al., 1996), and the crystal structure of the trimer solved (Schmidt et al., 2016). The molecular identity of the sigma-2 binding site has only recently been determined as TMEM97, a regulator of the sterol transporter NPC1 (Alon et al., 2017). Both proteins appear to be predominantly in the endoplasmic reticulum and, despite some evidence that progesterone and dimethyltryptamine bind the sigma-1 receptor (Fontanilla et al., 2009), are both considered orphan receptors. The sigma receptors have been implicated in a large number of apparently diverse conditions, including addiction, depression, pain, neurodegenerative conditions, cancer, and amyolateral sclerosis (among others), suggesting that their pharmacological regulation will yield useful drugs to treat several conditions.

Following the successful “Sigma-1 receptors as therapeutic targets” symposium, held at the Pharmacology 2017 meeting of the British Pharmacological Society, we organized a Research Topic entitled “Sigma Receptors” in *Frontiers in Pharmacology*. A total of 15 articles, consisting of 11 original papers and 4 review papers, has been published. Our Research Topic has been well received by the readership, with over 32,000 views to date. Here, we highlight the key outcomes of these publications.

Using a bibliographical approach to sigma receptor research, Romero and Portillo-Salido show how there has been an increase in publication numbers in this area. Focusing on the period 1992–2017, they identify highlights in sigma receptor research. Key findings include their cloning, the production of sigma-1 receptor knock-out mice and solving the crystal structure of the sigma-1 receptor. Their interesting analysis of the research landscape in this very dynamic field reveals numerous potentialities and collaborative networks. Furthermore, they show that ESTEVE, with interests in pain management and treatment of neurodegenerative disorders, is the industry leader of the field in publication number.

Vidal-Torres et al. show that S1RA (E-52862, MR309) alone is unable to elicit an antinociceptive effect by itself in the tail-flick acute pain assay in mice. However, in combination with opioids, a synergistic antinociceptive effect is observed. The enhanced opioid response is only observed following systemic or supraspinal administration of S1RA, but not following spinal administration. Additionally, loperamide, a peripherally restricted opioid, becomes effective as an antinociceptive agent in combination with S1RA. Therefore, both supraspinal and peripheral actions might account for the enhancement of opioid effects by sigma-1 antagonism.

Using an inflammatory pain model (periarticular inflammation induced by Complete Freund’s Adjuvant) in mice, Montilla-Garcia et al. show how the sigma-1 receptor antagonist S1RA acts synergistically with morphine to elicit an antinociceptive effect to reverse tactile allodynia. Importantly, S1RA was able to rescue the effect of morphine in tolerant mice following repeated injections of this opioid drug not only in tactile allodynia but also in pain-induced deficits in physical function (grip strength). These two studies point to that sigma-1 antagonists are promising tools as opioid adjuvants in chronic pain indications.

Cirino et al. and Bravo-Caparrós et al. provide further evidence that sigma receptors act directly as antinociceptive targets: Cirino et al. consider analgesic and anti-allodynic activities of two novel sigma ligands – a selective sigma-1 receptor antagonist, CM-304, and a non-selective sigma receptor ligand, AZ-66. Both are effective in a wide array of animal pain models: chronic nerve constriction injury; cisplatin neuropathy; acetic acid-induced writhing; formalin-induced inflammation; and the thermally-induced tail withdrawal tests. These results show that sigma-1 antagonists can have analgesic activity in the absence of exogenous opioid. Using sigma-1 receptor knock-out mice, Bravo-Caparrós et al. give evidence that this receptor is crucial in pain perception and relief using a spared nerve injury model in mice, where tibial and common peroneal branches of the sciatic nerve are ligated. The administration of S1RA decreases neuropathic cold, heat and tactile hypersensitivity. The effects of sigma-1 antagonism on heat and tactile hypersensitivity during neuropathic pain are indirectly mediated by the activation of the peripheral opioid receptors, which together with similar findings previously described during inflammatory hypersensitivity (Tejada et al., 2017), point to the relevance of sigma-1 receptors as a physiological modulator of the opioid system during pain conditions.

A common source of chronic pain is with the development of osteoarthritis. Using a mouse model, induced by an intrarticular injection of monosodium iodoacetate, Carcolé et al. show that S1RA is able to reverse hyperalgesia. Furthermore, S1RA treatment is able to prevent some of the profound behavioral changes induced by chronic pain, such as cognitive deficits (determined using a V-maze), and depressive-like states (determined using the forced-swim test) associated with osteoarthritis pain in mice.

The sigma-1 receptor is also described as a chaperone (Maurice and Su, 2009), and the knowledge on its interactome is increasingly growing. The review by Morales-Lázaro et al. gives further details about the interactions between the sigma-1 receptor and ion channels, bringing together details of how ligands for the sigma-1 receptor can regulate functional properties and the expression of some sodium, calcium, potassium, and TRP ion channels. The effects of agonists and antagonists at the sigma-1 receptor are discussed. Also focusing on the interactome of sigma-1 receptors, Cortés-Montero et al. show that sigma-1 receptors bind specific regions of TRP ankyrin member 1 (TRPA1), TRP vanilloid member 1 (TRPV1), and TRP melastatin member 8 (TRPM8) in a calcium sensitive manner. Agonists and antagonists are able to regulate interactions of the sigma-1 receptor with TRPA1, TRPM8, and TRPV1 in opposing fashions. This study adds significantly to our understanding about the mechanisms of action of sigma-1 receptors on nociception.

Other potential uses of sigma-1 receptor antagonists are in the treatment of cancers. Sigma-1 receptor antagonists show desirable properties, as they readily kill most tumor cell lines, while having minimal effect on most non-tumor cells (Spruce et al., 2004). A rigorous review by Oyer et al. brings together the information regarding the consequences of targeting sigma-1 and sigma-2 receptors in cancer. The consensus lies with sigma-1 receptor antagonists reducing proliferation. While the distinction between agonists and antagonists at the sigma-2 receptor remains uncertain, it appears that agonists at this receptor also possess antiproliferative properties. The pathways involved in sigma receptor signaling are logically presented and this paper provides an excellent guide to both established and naïve researchers in the sigma receptor field.

Adding new evidence on the potential use of sigma ligands as antineoplastics, Tesei et al. show the mixed sigma-1 receptor antagonist/sigma-2 receptor agonist RC-106 has anti-cancer properties in pancreatic cell lines. Treatment of cells with RC-106 drives apoptosis *via* upregulation of GRP78/BiP, ATF4, and CHOP mRNA expression levels, a common means of monitoring the ER unfolded protein response. With favorable pharmacokinetics and pancreas distribution, Tesei et al. propose RC-106 as a good candidate for further investigation *in vivo*.

In addition to the potential antineoplastic effects of sigma ligands, early data with *in vivo* localization of primary tumors and their metastases (Kawamura et al., 2005) in animal models have highlighted the value of imaging tools targeting the sigma receptors. Ludwig et al. further characterize (S)-[^18^F] fluspidine and identify its biological half-life and route of metabolism. The profile obtained *in vitro* and *in vivo* suggest (S)-[^18^F] fluspidine is suitable for PET imaging in humans.

In a review considering alcohol use disorder, Quadir et al. bring together data on the paucity of current treatments and the potential offered by sigma-1 receptor antagonists: antagonists reduce alcohol consumption, motivation to drink, and alcohol-seeking behavior. The use of knock-out mice verifies that sigma-1 receptors play a critical role in alcohol-mediated stimulant, motivational, and reward properties observed.

With so much interest in sigma receptors, it is key that the assays used to quantify receptor number in cells, and characterize ligand interactions are robust. Following on from a previous paper in which sigma-1 receptor binding to antagonists showed two affinity states in a GTP-dependent manner (Brimson et al., 2011), we now see a cautionary tale of why masking protocols, widely used for studying sigma-2 receptor interactions, should not be used. A common practice is to “mask” sigma-1 receptors with (+) pentazocine (or dextrallorphan) while using the pan-sigma ligand, [³H] DTG as radioligand. Abbas et al. demonstrate that saturation binding assays will permit DTG to bind sigma-1 receptors, hence over-estimating the sigma-2 receptor population. Equally, (+) pentazocine and dextrallorphan will bind to sigma-2 receptors and affect the apparent affinity of ligands for that receptor.

Remodeling the pharmacophore for the sigma-1 receptor is also undertaken: using the crystal structure (Schmidt et al., 2016) and binding data from over 25,000 ligands, Pascual et al. refine interaction predictions which can be used to design novel drugs targeting this receptor.

In their review, Vavers et al. lead us through the first identification of an allosteric regulator of sigma-1 receptors: phenytoin, notorious for its zero-order kinetics and induction of cytochrome P450 enzymes, is also a positive allosteric modulator (PAM) of the sigma-1 receptor. While the antiseizure activity of phenytoin is not reversed by BD-1047, a sigma-1 receptor antagonist, the activity of many other PAMs is reversed. Today, several PAMs have been identified acting at the sigma-1 receptor and are shown to exert potent *in vivo* effects, such as improving memory and bearing antidepressant activity.

With such a variety of very important results, articles presented in this topic very nicely illustrate the dynamism and importance of present research in sigma receptors. With such interesting recent developments in the field, we hope we can bring an update in the near future as the research contained herein is brought to fruition.

## Author Contributions

All authors contributed to the article and approved the submitted version.

## Conflict of Interest

The authors declare that the research was conducted in the absence of any commercial or financial relationships that could be construed as a potential conflict of interest.
